# We need to optimize piperacillin-tazobactam dosing in critically ill patients—but how?

**DOI:** 10.1186/s13054-016-1348-8

**Published:** 2016-06-06

**Authors:** Menino Osbert Cotta, Jason A. Roberts, Jeffrey Lipman

**Affiliations:** Royal Brisbane and Women’s Hospital, Brisbane, Australia; School of Pharmacy, The University of Queensland, Brisbane, Australia; School of Medicine, The University of Queensland, Brisbane, Australia; Queensland University of Technology, Brisbane, Australia; The University of Witwatersrand, Johannesburg, South Africa; Department of Intensive Care, Royal Brisbane and Women’s Hospital, Herston, Queensland Australia

Zander et al. [1] have recently conducted a prospective observational study to describe the variability of piperacillin (PIP) concentrations and target attainment in a heterogeneous cohort of 60 critically ill patients. An intermittent bolus dosing regimen of piperacillin-tazobactam (PIP-TAZ) was used 4.5 g three times daily (TID) or twice daily (BID), depending on renal function.

As PIP-TAZ is largely renally excreted, it was unsurprising that the investigators found that no patient within the highest quartile of creatinine clearance (CrCl) attained specified pharmacokinetic/pharmacodynamic (PK/PD) targets (trough concentrations ≥22.5 mg/L) on days 1 or 4. These results are consistent with previous data showing that ‘augmented renal clearance’ (ARC), defined as a CrCl ≥130 mL/min [2], is frequently being associated with subtherapeutic PIP concentrations, even when dosing PIP-TAZ four times daily (QID) [2, 3]. Of more interest, however, was that the investigators found that 0 % and 55 % of patients with a CrCl >65 mL/min and 30–65 mL/min, respectively, attained the target PIP trough concentrations [1]. This is a very important finding as it highlights that initiating TID PIP-TAZ dosing as a blanket strategy in critically ill patients with ‘normal’ renal function or even mild to moderate renal impairment will not reliably attain PK/PD targets. In line with these findings, and perhaps reflective of the ongoing challenges with dose optimization in this group of patients, previous work has shown QID dosing of PIP-TAZ to be insufficient in achieving free PIP concentrations that are four times the target minimum inhibitory concentration (MIC) at 50 % of the dosing interval (50%*f*T_>4xMIC_), even in patients not displaying ARC [4, 5]. Solutions for target non-attainment of PIP-TAZ in intensive care unit (ICU) patients include use of prolonged infusions and therapeutic drug monitoring (TDM).

Administering PIP-TAZ as a prolonged infusion—that is, either administering the antibiotic over half the dosing interval (e.g., over 3 h if given QID), or administering the total daily dose as a continuous infusion over 24 h after an initial loading dose—may help overcome subtherapeutic PIP concentrations in the critically ill [6]. There are now observational studies as well as randomized controlled trial (RCT) data that show increased likelihood of PIP concentrations being maintained above the MIC of pathogens using the prolonged infusion strategy [7–9]. Furthermore, there were higher rates of clinical cure associated with PIP-TAZ administered as a continuous infusion in the RCTs [7, 9]. Both of the RCTs included patients with mild to moderate renal impairment, and so, coupled with the results from the study by Zander et al. [1] showing PK/PD target attainment being problematic in these groups of patients via intermittent bolus dosing, the use of prolonged infusions may be a simple strategy that ICUs can employ. Notably, however, prolonged infusions may still not achieve therapeutic targets in certain groups of patients, such as those displaying ARC [10]. In these circumstances, dose up-titration may also be advisable, but can only be comfortably performed with use of TDM.

To this end, a major challenge in determining optimal PIP-TAZ dosing for individual critically ill patients is the high inter-patient variability in serum PIP trough concentrations. Zander and investigators noted a 123-fold to >1785-fold range of PIP trough concentrations among their study patients, which was more pronounced in patients with higher creatinine clearances than those with severely impaired renal function [1]. Other investigators have also shown high inter-patient variability with PIP-TAZ dosing [11, 12], albeit in studies with smaller sample sizes of more homogeneous groups of critically ill patients. From these PK data, as well as due to pathogen-related factors such as decreasing antibiotic susceptibilities, as reflected by higher MICs, it is becoming evident that an individualized dosing approach needs to be adopted in order to maximize PIP-TAZ efficacy. Given this, there is significant scope for TDM of PIP-TAZ as well as other beta-lactam antibiotics in the ICU. Monitoring of trough concentrations at a steady state (usually after 3 to 4 doses) is generally recommended to see whether PK/PD targets are being met [13]. Earlier measurement should be preferred where dosing can be optimized using Bayesian dosing software [14]. Additionally, measurement of the ‘free’ or unbound PIP concentrations, rather than ‘total’ concentrations as described by Zander et al. [1], is advised. Although PIP is not highly protein bound (typically 30 %), differences in protein binding among patients, particularly those in hypoalbuminemic states, as well as alterations in protein binding during assay preparation, means there is much guess work determining PK/PD targets when using ‘total’ rather than ‘unbound’ antibiotic concentrations [15].

Interestingly, although Zander and colleagues found intra-patient variability to be less than inter-patient variability in their study cohort, the coefficient of variations (CV) for intra-patient PIP concentration variability was still wide, ranging from 6.4 to 129 % (median of 30 %) [1]. The heterogeneity of the study population most likely contributed to this wide intra-patient variability; however, the median CV for intra-patient variability is comparable to the median CV of 40 % reported by Carlier et al. [11], and reflects the key message that consistent dosing of PIP-TAZ does not necessarily result in consistent PIP concentrations throughout the period of therapy. This suggests that the use of merely ‘once-off’ TDM may be inadequate in determining if PK/PD targets are being attained consistently throughout therapy duration. We believe this study and previous work make the case for more frequent, perhaps even daily, use of TDM to ensure ongoing efficacy of PIP-TAZ in the critically ill patient [11].

## Conclusions

Zander and colleagues have provided new information on target attainment of PIP concentrations in a heterogeneous group of critically ill patients. Among patients without severe renal dysfunction, there are concerns of not reaching PK/PD targets using traditional TID dosing of PIP-TAZ. There also is high inter- and intra-patient variability in PIP concentrations. Alternative dosing strategies that optimize PIP-TAZ therapy are needed and may include the use of prolonged infusions. Importantly, there is strong rationale for the use of regular TDM to be the cornerstone for individualizing PIP-TAZ therapy and maximize efficacy.

## Abbreviations

ARC, augmented renal clearance; BID, twice daily; CrCl, creatinine clearance; CV, coefficient of variations; ICU, intensive care unit; MIC, minimum inhibitory concentration; PIP, piperacillin; PIP-TAZ, piperacillin-tazobactam; PK/PD, pharmacokinetic/pharmacodynamic; QID, four times daily; RCT, randomized controlled trial; TDM, therapeutic drug monitoring; TID, three times daily; 50%*f*T_>4xMIC_, free drug concentrations four times the target minimum inhibitory concentration at 50 % of the dosing interval
